# Retraction: MicroRNA-506 inhibits rheumatoid arthritis fibroblast-like synoviocytes proliferation and induces apoptosis by targeting TLR4

**DOI:** 10.1042/BSR-2018-2500_RET

**Published:** 2021-07-28

**Keywords:** fibroblast-like synoviocytes, miR-506, Rheumatoid arthritis

This article is being retracted from *Bioscience Reports* at the request of the Editorial Board following receipt of a notification from a reader, alerting the Editorial Office to duplications in the Western blots of Figure 2D and flow-cytometry in Figure 3A which have been published in several other publications by different authors.

The authors contacted the Editorial Office to request a Correction however, given the extent of the issues raised, the Editorial Board stand by the decision to retract the article. The authors disagree to the Retraction.

